# A New Self-Calibration and Compensation Method for Installation Errors of Uniaxial Rotation Module Inertial Navigation System

**DOI:** 10.3390/s22103812

**Published:** 2022-05-17

**Authors:** Meng Niu, Hongyu Ma, Xinglin Sun, Tiantian Huang, Kaichen Song

**Affiliations:** 1 College of Biomedical Engineering and Instrument Science, Zhejiang University, Hangzhou 310027, China; nium@zju.edu.cn (M.N.); tthuang@zju.edu.cn (T.H.); 2 School of Aeronautics and Astronautics, Zhejiang University, Hangzhou 310027, China; 3170101710@zju.edu.cn (H.M.); kcsong@zju.edu.cn (K.S.)

**Keywords:** inertial navigation system, uniaxial rotation module, self-calibration, installation errors, information redundancy

## Abstract

Calibration and compensation techniques are essential to improve the accuracy of the strap-down inertial navigation system. Especially for the new uniaxial rotation module inertial navigation system (URMINS), replacing faulty uniaxial rotation modules introduces installation errors between modules and reduces navigation accuracy. Therefore, it is necessary to calibrate these systems effectively and compensate for the installation error between modules. This paper proposes a new self-calibration and compensation method for installation errors without additional information and equipment. Using the attitude, velocity, and position differences between the two sets of navigation information output from URMINS as measurements, a Kalman filter is constructed and the installation error is estimated. After URMINS is compensated for the installation error, the average of the demodulated redundant information is taken to calculate the carrier’s navigation information. The simulation results show that the proposed method can effectively assess the installation error between modules with an estimation accuracy better than 5”. Experimental results for static navigation show that the accuracy of heading angle and positioning can be improved by 73.12% and 81.19% after the URMINS has compensated for the estimated installation errors. Simulation and experimental results further validate the effectiveness of the proposed self-calibration and compensation method.

## 1. Introduction

The superiority of a strap-down inertial navigation system (SINS) is that no external reference is needed to determine the vehicle’s attitude, velocity, or position, and no information is transmitted to the environment [1]. Therefore, it is extensively used in vehicles, submarines, missiles, aircraft and ships [2,3,4,5]. A global positioning system (GPS) is usually integrated with SINS to improve the carrier’s navigation and positioning accuracy. However, GPS is unavailable in some environments, such as underwater navigation and indoor localization [6]. Compared with the GPS, the SINS has the advantages of good concealment and strong anti-interference capability and can be applied in GPS-denied environments such as underwater navigation [7,8]. However, the drawback of SINS is that navigation errors accumulate with time owing to the drift-bias error of internal sensors such as gyroscope and accelerometer, which deteriorates the navigation accuracy of SINS [9]. Since the carrier may work in a covert environment, no other information is available to fuse with the SINS to suppress errors. Furthermore, if sensors in the SINS fail, these failed sensors must be replaced and maintained for the system to function correctly. However, reinstalling new sensors introduces errors into the system. The traditional method of calibrating the system for various errors is to take the inertial navigation system back to the laboratory for recalibration [10], which is inconvenient and wastes time. Therefore, the self-calibration and self-compensation techniques of errors in the SINS are of great significance for improving the performance of SINS [11,12].

Nowadays, rotation modulation technology is the primary method to realize the self-compensation of errors in the SINS, which can suppress the bias drift and other slowly changing errors of inertial sensors by periodic rotation [13,14]. The inertial sensors are rotated around one axis or multiple axes [15,16,17]. The uniaxial rotation modulated inertial navigation system (RMINS) has been widely studied and applied [18]. However, the inertial sensor’s constant bias along the rotational axis cannot be suppressed and still influences the navigation accuracy of SINS [19]. The inertial sensors’ constant errors are compensated in bi-axis and tri-axis RMINS, but the reliability of the RMINS is reduced due to the complex mechanics [20,21,22]. To avoid the complex multi-axis mechanism and enhance the accuracy and reliability of SINS, a novel triple rotary unit strap-down inertial navigation system (TRUSINS) was proposed. Each rotating unit of TRUSINS is uniaxial rotation, and the positioning accuracy of TRUSINS is improved by ten times compared to SINS [23]. It can be seen that the application of uniaxial rotation and data redundancy has a great development prospect in improving the accuracy of SINS. To improve the accuracy and configuration flexibility of SINS and simplify the design complexity of rotating shafting, a uniaxial rotation module inertial navigation system (URMINS) composed of several single-axis rotating modules is proposed in this paper. The structure of URMINS is described in Section 2 of this article. This navigation system can be applied in GPS-denied environments, such as outdoor navigation of vehicles, ships, and autonomous underwater vehicles.

However, with structures such as TRUSINS and URMINS, installation errors between modules have a detrimental effect on the system’s accuracy and must be compensated for [24]. The proposed methods used to calibrate errors of SINS are generally divided into direct and indirect methods. The direct calibration method uses the turntable to provide accurate reference information to SINS and estimates various error parameters in the system model by comparing the output of SINS with the reference information. For example, reference [25] used a single-axis turntable rotation to generate a reference angular rate for calibrating the gyro error. However, the calibration accuracy is related to the precision of the turntable [26]. The indirect calibration methods are used to determine error parameters by observing vehicle’s attitude, velocity or position errors. For example, reference [16] proposed an estimation method for calibrating nonorthogonal angle in dual-axis rotational INS, which takes attitude, velocity and altitude errors output by SINS as measurement values and assumes that the carrier is in a stationary state during the calibration process. Another study ([27]) based on the static environment used the attitude angle difference as the measurement value of a Kalman filter to estimate the error parameter to improve the attitude output accuracy. Once the carrier is in motion, the output of SINS contains the carrier’s velocity, attitude, and position information. It is difficult to estimate the error without the assistance of other sensors.

Current calibration methods are usually based on laboratory turntables or external information-generated reference signals to calibrate installation errors in the SINS [28]. In recent years, to calibrate the error of inertial sensors accurately, some novel calibration methods based on machine learning have also been widely studied [29,30]. However, these methods generally use high-precision data to train a network model and then apply the model to low-precision data to improve performance. That is to say, the novel calibration method based on machine learning still needs some external information assistance to obtain the error model of the system or sensor. Therefore, the limitation of the existing approach is that it is difficult to estimate the installation error caused by module replacement under dynamic conditions without external information. For calibrating the SINS’s error in a dynamic environment, a method was proposed to estimate the installation error by using the difference in navigation information output by two sets of SINS installed on the same carrier [31,32,33]. Nevertheless, the uniaxial rotation module (URM) consists of only two gyroscopes and two accelerometers, and a single module cannot output navigation information. Therefore, calibration methods for dynamic conditions cannot be directly applied to the URMINS to estimate installation errors caused by the replacement of a faulty module.

This paper proposes a new method for self-calibration and compensation of URMINS installation errors. Compared with existing methods, this proposed method can calibrate the installation error of URMINS in a dynamic environment without additional information sources or external devices. This approach uses the two sets of attitude, velocity, and position differences from URMINS as measurements. The Kalman filter is applied to estimate the installation error, given the linear URMINS error model and measurement error. The advantage of the Kalman filter is that it uses all measurements, regardless of their accuracy, to estimate system state values by properly weighting these measurements. However, we do not think that the Kalman filter is better than other filters when estimating installation errors. Many researchers have studied improvement algorithms of the traditional Kalman filter so that the performance of the Kalman filter has been improved [34].

The motivation behind and contributions of this paper are summarized as follows. First, to improve the navigation accuracy and system configuration flexibility of pure SINS and simplify the design of the rotating shaft, this paper proposes a URMINS composed of multiple URMs. Each URM can demodulate measurements from two gyroscopes and two accelerometers, with inertial sensor measurements oriented perpendicular to the axis of rotation. Second, to realize the replacement and maintenance of faulty URMs in a dynamic environment without relying on external equipment, this paper proposes a self-calibration and compensation method for the installation error caused by replacing faulty URMs. Using the difference between the two sets of navigation information output by URMINS as the measurement value of the Kalman filter, the installation error is stimulated by the movement of the carrier in the dynamic environment and is estimated. An indexing scheme is designed to suppress the inertial sensor’s constant and slowly changing errors. Installation errors are compensated for during the demodulation of the URMINS information. Both simulation and experimental results show that the method can calibrate the URMINS installation errors and that the navigation accuracy is improved after the installation error are compensated significantly.

The rest of this paper is organized as follows. In Section 2, the configuration of URMINS is presented, and the installation error model is established and analyzed; In Section 3, the self-calibration and compensation scheme are given. Simulation results are provided in Section 4 to prove the method’s validity. Experimental results and analysis are presented in Section 5. Finally, our conclusions are summarized in Section 6.

## 2. Configuration of URMINS and Installation Error Modeling

### 2.1. Configuration of URMINS

**Figure 1:**
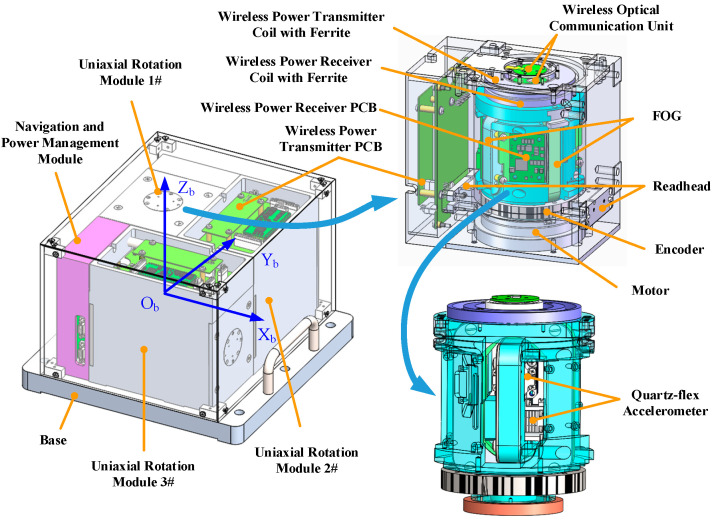
The mechanical structure of URMINS.

This system includes three same URMs and one navigation and power management module, and each module is mounted on the base. The mechanical structure of URMINS is shown in Figure 1. The rotary axes of the URM are perpendicular to each other, forming a Cartesian coordinate system. Each URM comprises two fiber optic gyroscopes (FOGs), two quartz-flexible accelerometers, a frameless torque motor, a RESM angle encoder, two read-heads, a wireless communication unit, and a wireless power transmission unit. The inertial sensors in each rotation framework rotate around the corresponding axis. The wireless data communication (wireless optical communication unit) and wireless power transmission are used in each module to avoid wire entanglement and friction of slip rings. The CAN bus is used for data transmission between modules and between each module and the navigation and power management module. Since each module has the same structure, the modules are interchangeable and convenient for replacement and maintenance.

### 2.2. The Installation Error Modeling and Analysis

Next, some proper coordinate frames relevant to self-calibration, navigation, and mutual transformations are discussed.

The *i*-frame is the earth-centered inertial frame. The Earth-centered Earth-fixed frame is the *e*-frame which rotates along with the earth. The navigation frame, named *n*-frame, its axes aligned with the east, north, and up directions. The body frame is referred to as the *b*-frame. The *bi*-frame (i=1,2,3) represents the orthogonal coordinate system obtained after calibration in the laboratory turntable for each module. *Bi*-frame (i=1,2) represents the equivalent body coordinate system of the combined strap-down inertial navigation system (CSINS), including CSINS1 and CSINS2. The *s*0-frame is the rotation coordinate frame under the ideal installation conditions at the initial moment. The *s*-frame is the rotation coordinate frame under the ideal installation conditions at any time. The *g*-frame and *a*-frame are the actual installation frames for the gyroscope and accelerometer, respectively. Here, the *a*-frame and *g*-frame are nonorthogonal coordinates, while the other frames are orthogonal. The coordinate frames mentioned above belong to the right-hand coordinate system.

To describe the transformation between two coordinate systems clearly, the transformation matrix $C_{t}^{k}$ *(*t=a,g,s,s0,b; k=s,s0,b,n,Bi*)* is introduced to represent the transformation from *t*-frame to *k*-frame. Since the internal structure of each module is the same, the direction of the rotation axis between modules is different. Therefore, take URM 1# as an example, and continue to analyze the installation error. In the *b*1-frame, the measured value of the accelerometer is $f^{b1}$.

(1) $$f^{b1}=C_{s0}^{b1}C_{s}^{s0}C_{a}^{s}f^{a}$$

And then, the transformation matrices in Equation (1) are written as follows.

(2) $$C_{a}^{s}=\left[\begin{array}{ccc} 1 & -\beta_{xz}^{1} & \beta_{xy}^{1}\\ \beta_{yz}^{1} & 1 & -\beta_{yx}^{1}\\ -\beta_{zy}^{1} & \beta_{zx}^{1} & 1 \end{array}\right]\;$$

(3) $$C_{s}^{s0}=\left[\begin{array}{ccc} cos\theta_{1} & -sin\theta_{1} & 0\\ sin\theta_{1} & cos\theta_{1} & 0\\ 0 & 0 & 1 \end{array}\right]\;$$

(4) $$C_{s0}^{b1}=\left[\begin{array}{ccc} 1 & -\mu_{z}^{1} & \mu_{y}^{1}\\ \mu_{z}^{1} & 1 & -\mu_{x}^{1}\\ -\mu_{y}^{1} & \mu_{x}^{1} & 1 \end{array}\right]\;$$

In the transformation matrix $C_{a}^{s}$, the installation error angles $\beta_{zy}^{1}$ and $\beta_{zx}^{1}$ are zero since no sensor is mounted along the $Z_{a}^{1}$-axis. The rotation angle of the motor is indicated by the symbol $\theta_{i}\;\left(i=1,2,3\right)$, where *i* is the module number. The vector $f^{a}$ represents the measurement of the accelerometer installed in URM 1# in the *a*-frame, where there exists $f^{a}={\left[f_{x1}^{a}\;f_{y1}^{a}\;0\right]}^{T}$.

**Figure 2:**
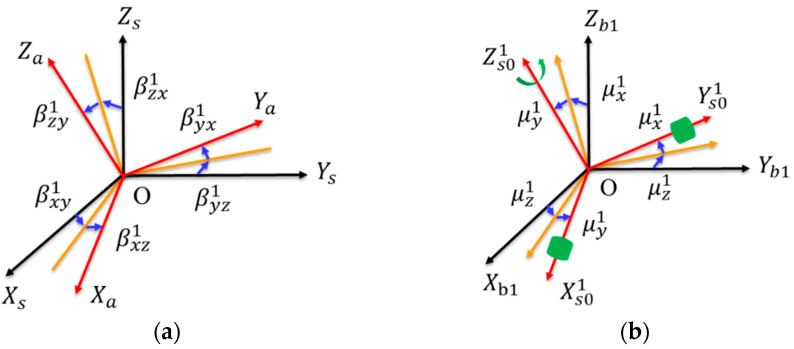
(**a**) Installation errors from the *a*-frame to the *s*-frame; (**b**) the installation errors of URM 1# from the *s*0-frame to the *b*1-frame.

There are two types of installation errors in the URMINS, one is within the URM, and the other is between modules. The installation error inside the URM is introduced by the transformation from a nonorthogonal coordinate system, such as *a*-frame and *g*-frame, to an orthogonal coordinate system, such as *b*1-frame, *b*2-frame, and *b*3-frame. Take the transformation of the accelerometer’s frame as an example. The modeling of installation errors from the *a*-frame to the *s*-frame is shown in Figure 2a. Here, the installation error angle is represented by $\beta_{ij}^{k}$ *(i, j* = x,y,z; *k* = 1, 2, 3*)*, representing the installation error angle obtained by rotating the *i*-axis of the *a*-frame around the *j*-axis of the *s*-frame. The installation error from the *s*0-frame to the *b*-frame is denoted by the symbol $\mu_{k}^{i}\;$*(*i=1, 2, 3; k=x,y,z*)*, where *i* is the module number, and *k* represents the axis. Installation errors modeling of URM 1# from the *s*0-frame to *b*1-frame is shown in Figure 2b. When each module is calibrated on the same base, the *b*1-frame, the *b*2-frame, and the *b*3-frame coincide with the *b*-frame. Green cylinders represent sensors, including a gyroscope and an accelerometer. In the *s*0-frame, sensors are installed along the *X*-axis and the *Y*-axis. The *Z*-axis is the rotation axis without installing any sensors. Installation errors within the URM 2# and URM 3# are defined in the same way as URM 1#, and the rotation axes are the *Y*-axis and the *X*-axis, respectively.

The accelerometers’ output errors of the *X*-axis and *Y*-axis in the *b*-frame due to the absence of the *Z*-axis are shown in Equation (5). Here, we only consider the primary term of the installation error.

(5) $$\begin{array}{c} \delta f_{x1}^{b1}=-\left(\beta_{yz}^{1}+\mu_{z}^{1}\right)sin\theta_{1}f_{x1}^{a}-\left(\beta_{xz}^{1}+\mu_{z}^{1}\right)cos\theta_{1}f_{y1}^{a}+\left(\mu_{y}^{1}+\beta_{yx}^{1}sin\theta_{1}+\beta_{xy}^{1}cos\theta_{1}\right)f_{z}^{a}\\ \delta f_{y1}^{b1}=\left(\beta_{yz}^{1}+\mu_{z}^{1}\right)cos\theta_{1}f_{x1}^{a}-\left(\beta_{xz}^{1}+\mu_{z}^{1}\right)sin\theta_{1}f_{y1}^{a}+\left(-\mu_{x}^{1}+\beta_{xy}^{1}sin\theta_{1}-\beta_{yx}^{1}cos\theta_{1}\right)f_{z}^{a} \end{array}$$

Similarly, the accelerometers’ output errors of the URM 2# and URM 3# can be deduced as follows. The derivation of Equations (6) and (7) is shown in Appendix A.

(6) $$\begin{array}{c} \delta f_{x2}^{b2}=-\left(\beta_{zy}^{2}+\mu_{y}^{2}\right)sin\theta_{2}f_{x2}^{a}+\left(-\mu_{z}^{2}+\beta_{zx}^{2}sin\theta_{2}-\beta_{xz}^{2}cos\theta_{2}\right)f_{y}^{a}+\left(\beta_{xy}^{2}+\mu_{y}^{2}\right)cos\theta_{2}f_{z2}^{a}\\ \delta f_{z2}^{b2}=-\left(\beta_{zy}^{2}+\mu_{y}^{2}\right)cos\theta_{2}f_{x2}^{a}+\left(\mu_{x}^{2}+\beta_{xz}^{2}sin\theta_{2}+\beta_{zx}^{2}cos\theta_{2}\right)f_{y}^{a}-\left(\beta_{xy}^{2}+\mu_{y}^{2}\right)sin\theta_{2}f_{z2}^{a} \end{array}$$

(7) $$\begin{array}{c} \delta f_{y3}^{b3}=\left(-\mu_{z}^{3}+\beta_{zy}^{3}sin\theta_{3}-\beta_{yz}^{3}cos\theta_{3}\right)f_{x}^{a}-\left(\beta_{zx}^{3}+\mu_{x}^{3}\right)sin\theta_{3}f_{y3}^{a}-\left(\beta_{yx}^{3}+\mu_{x}^{3}\right)cos\theta_{3}f_{z3}^{a}\\ \delta f_{z3}^{b3}=\left(-\mu_{y}^{3}+\beta_{yz}^{3}sin\theta_{3}-\beta_{zy}^{3}cos\theta_{3}\right)f_{x}^{a}+\left(\beta_{zx}^{3}+\mu_{x}^{3}\right)cos\theta_{3}f_{y3}^{a}-\left(\beta_{yx}^{3}+\mu_{x}^{3}\right)sin\theta_{3}f_{z3}^{a} \end{array}$$

**Figure 3:**
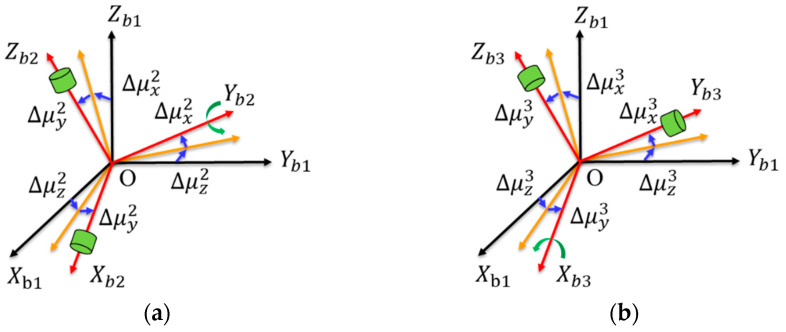
(**a**) The installation error of URM 2# from the *b*2-frame to the *b*1-frame; (**b**) the installation error of URM 3# from the *b*3-frame to the *b*1-frame.

For the URM 1#, installation errors $\beta_{xy}^{1}$, $\beta_{yx}^{1}$,$\;\mu_{x}^{1},$ $\varphi_{y}^{1}$, $\beta_{yz}^{1}+\mu_{z}^{1}$, $\beta_{xz}^{1}+\mu_{z}^{1}$ can be calibrated in the laboratory, and the non-orthogonal installation error $\beta_{ij}^{k}$ will not be changed by module reinstallation, so this paper only considers the variation of orthogonal installation error $\mu_{k}^{i}$ between modules. Here, the variation of the installation error angle introduced by the reinstallation of the module is defined as $\Delta \mu_{k}^{i}\;\left(i=1,\;2,\;3;\;k=x,y,z\right)$, and the installation error angle between *b*2-frame and *b*3-frame and *b*1-frame is shown in Figure 3a–b.

Since the installation error coupling terms $\beta_{yz}^{1}+\mu_{z}^{1}$ and $\beta_{xz}^{1}+\mu_{z}^{1}$ cannot be separated, they are treated as one term. The error term related to $f_{z}^{a}$ in Equation (5) is not compensated in a single module since there is no sensor mounted in the direction of the rotation axis. Hence, the absence of this measurement $f_{z}^{a}$ in a single module increases the navigation error. Similarly, the installation errors in URM 2# and URM 3# are the same as the analysis method in URM 1#. Therefore, it is necessary to study the self-calibration and compensation method of the installation error $\Delta \mu_{k}^{i}$, and a scheme will be given in Section 3.

## 3. Self-Calibration and Compensation Scheme

### 3.1. Self-Calibration Scheme

This system comprises three URMs, including six gyroscopes and six accelerometers. Two sets of inertial navigation systems, CSINS1 and CSINS2, can be formed using redundant information. There are four ways to configure two groups of inertial navigation systems when all sensors’ output information is applied. Using the difference in the navigation information of the two inertial navigation systems as a measure of the Kalman filter, the installation error caused by the module replacement is estimated.

#### 3.1.1. Design of Kalman Filter

The angular velocity and specific force measured by the sensors perpendicular to the rotary axis are demodulated to the *b*-frame and output through the URM. Therefore, six angular velocities and specific forces from three URMs can be obtained in the *b*-frame, and there are two sets of measurements along with the directions $X_{b}$, $Y_{b}$, and $Z_{b}$. Here, the CSINS1 consists of $X_{b1}$, $Y_{b1}$ of URM 1#, and $Z_{b3}$ of URM 3#, while $X_{b2}$, $Z_{b2}$ of URM 2#, and $Y_{b3}$ of URM 3# constitute the CSINS2. The two sets of CSINS calculate and output the navigation information. The difference in navigation information of CSINS1 and CSINS2 is used as the measurement value to estimate the installation errors between modules. Assuming that the *b*-frame overlapped with the *b*1-frame of URM 1#, the installation errors $\Delta \mu_{x}^{1}$, $\Delta \mu_{y}^{1}$ and $\Delta \mu_{z}^{1}$ are zero.

The state variable ***X*** is as follows.

(8) $$X={\left[\phi_{E12}\;\phi_{N12}\;\phi_{U12}\;\delta V_{E12}\;\delta V_{N12}\;\delta L_{12}\;\delta \lambda_{12}\;\Delta \mu_{x}^{2}\;\Delta \mu_{y}^{2}\;\Delta \mu_{z}^{2}\;\Delta \mu_{x}^{3}\;\Delta \mu_{y}^{3}\;\Delta \mu_{z}^{3}\right]}^{T}\;$$

The difference between the misalignment angles of CSINS1 and CSINS2 is written as $\phi_{12}={\left[\phi_{E12}\;\phi_{N12}\;\phi_{U12}\right]}^{T}=\phi_{1}-\phi_{2}$. The difference between the east and north velocity errors of CSINS1 and CSINS2 is noted as $\delta V_{12}={\left[\delta V_{E12}\;\delta V_{N12}\right]}^{T}=\delta V_{1}-\delta V_{2}$. The difference between the position errors of CSINS1 and CSINS2 is written as $\delta p_{12}={\left[\delta L_{12}\;\delta \lambda_{12}\right]}^{T}=\delta p_{1}-\delta p_{2}$. The symbol $∆\mu_{k}^{2}\;$*(*k=x,y,z*)* represents the installation error angle between URM 2# and URM 1#. The symbol $∆\mu_{k}^{3}\;$*(*k=x,y,z*)* represents the installation error angle between URM 3# and URM 1#.

The attitude error equations are as follows.

(9) $${\dot{\phi }}_{E12}=\left(w_{U}+\frac{V_{E}tanL}{R_{N}+h}\right)\phi_{N12}-\left(w_{N}+\frac{V_{E}}{R_{N}+h}\right)\phi_{U12}-\frac{1}{R_{M}+h}\delta V_{N12}-\delta w_{E12}^{n}$$

(10) $${\dot{\phi }}_{N12}=-\left(w_{U}+\frac{V_{E}tanL}{R_{N}+h}\right)\phi_{E12}-\frac{V_{N}}{R_{M}+h}\phi_{U12}+\frac{1}{R_{N}+h}\delta V_{E12}-w_{U}\delta L_{12}-\delta w_{N12}^{n}$$

(11) $${\dot{\phi }}_{U12}=\left(w_{N}+\frac{V_{E}}{R_{N}+h}\right)\phi_{E12}+\frac{V_{N}}{R_{M}+h}\phi_{N12}+\frac{tanL}{R_{N}+h}\delta V_{E12}+\left(w_{N}+\frac{V_{E}sec^{2}L}{R_{N}+h}\right)\delta L_{12}-\delta w_{U12}^{n}$$

The east and north velocity error equations are shown below.

(12) $$\delta {\dot{V}}_{E12}=-f_{U}\phi_{N12}+f_{N}\phi_{U12}+\frac{V_{N}tanL}{R_{N}+h}\delta V_{E12}+(2w_{U}+\frac{V_{E}tanL}{R_{N}+h})\delta V_{N12}+\left(2V_{N}w_{N}+\frac{V_{E}V_{N}sec^{2}L}{R_{N}+h}\right)\delta L_{12}+\delta f_{E12}^{n}$$

(13) $$\delta {\dot{V}}_{N12}=f_{U}\phi_{E12}-f_{E}\phi_{U12}-2(w_{U}+\frac{V_{E}tanL}{R_{N}+h})\delta V_{E12}-\left(2V_{E}w_{N}+\frac{V_{E}^{2}sec^{2}L}{R_{N}+h}\right)\delta L_{12}+\delta f_{N12}^{n}$$

The positional error equations for longitude and latitude are as follows.

(14) $$\delta {\dot{L}}_{12}=\frac{1}{R_{M}+h}\delta V_{N12}$$

(15) $$\delta {\dot{\lambda }}_{12}=\frac{secL}{R_{N}+h}\delta V_{E12}+\frac{V_{E}tanLsecL}{R_{N}+h}\delta L_{12}$$

The symbol $w_{ie}^{n}$ is the earth’s rotation velocity projection under the *n*-frame, and its expression is $w_{ie}^{n}={\left[w_{E}\;w_{N}\;w_{U}\right]}^{T}$. $R_{M}$ and $R_{N}$ are the curvature radius of the meridian and prime vertical, respectively. The symbol $f_{ia}^{n}$ is the specific force measured by the accelerometer in the *n*-frame, and its expression is $f_{ia}^{n}=C_{b}^{n}f_{ia}^{b}={\left[f_{E}\;f_{N}\;f_{U}\right]}^{T}$. The symbol $\delta w_{12}^{n}$ represents the difference in measurement error under the *n*-frame caused by gyroscopes of the two CSINSs, and its expression is $\delta w_{12}^{n}={\left[\delta w_{E12}^{n}\;\delta w_{N12}^{n}\;\delta w_{U12}^{n}\right]}^{T}$. The symbol $\delta f_{12}^{n}$ represents the difference in measurement error under the *n*-frame caused by the accelerometer measuring the assembly of the two CSINSs, and its expression is $\delta f_{12}^{n}={\left[\delta f_{E12}^{n}\;\delta f_{N12}^{n}\;\delta f_{U12}^{n}\right]}^{T}$. The specific expressions of the difference in sensor measurement error are shown in Equations (16) and (17).

(16) $$\delta w_{12}^{n}=C_{B1}^{n}\delta w_{1}^{B1}-C_{B2}^{n}\delta w_{2}^{B2}\;$$

(17) $$\delta f_{12}^{n}=C_{B1}^{n}\delta f_{1}^{B1}-C_{B2}^{n}\delta f_{2}^{B2}\;$$

Take the measurement error of the accelerometer as an example. It can be obtained from Equations (5)–(7). Furthermore, the measurement error of the accelerometer caused by CSINS1 and CSINS2 are presented as $\delta f_{1}^{B1}={\left[\delta f_{x1}^{b}\;\delta f_{y1}^{b}\;\delta f_{z3}^{b}\right]}^{T}$ and $\delta f_{2}^{B2}={\left[\delta f_{x2}^{b}\;\delta f_{y3}^{b}\;\delta f_{z2}^{b}\right]}^{T}$. They are obtained from Equations (18) and (19). Here, there is $\delta f_{x1}^{b}=\delta f_{y1}^{b}=0$.

(18) $$\begin{array}{c} \delta f_{x2}^{b}=-∆\mu_{y}^{2}sin\theta_{2}f_{x2}^{a}-∆\mu_{z}^{2}f_{y}^{a}+∆\mu_{y}^{2}cos\theta_{2}f_{z2}^{a}\\ \delta f_{z2}^{b}=-∆\mu_{y}^{2}cos\theta_{2}f_{x2}^{a}+∆\mu_{x}^{2}f_{y}^{a}-∆\mu_{y}^{2}sin\theta_{2}f_{z2}^{a}\; \end{array}$$

(19) $$\begin{array}{c} \delta f_{y3}^{b}=-∆\mu_{z}^{3}f_{x}^{a}-∆\mu_{x}^{3}sin\theta_{3}f_{y3}^{a}-∆\mu_{x}^{3}cos\theta_{3}f_{z3}^{a}\\ \delta f_{z3}^{b}=-∆\mu_{y}^{3}f_{x}^{a}+∆\mu_{x}^{3}cos\theta_{3}f_{y3}^{a}-∆\mu_{x}^{3}sin\theta_{3}f_{z3}^{a}\; \end{array}$$

In theory, there is $C_{B1}^{n}=C_{B2}^{n}$. The missing information in the third axis of each URM is determined by the average of the other two modules, whose expressions are shown in Equations (20)–(22). This method is also applicable to compensation installation errors of URMINS.

(20) $$f_{x}^{a}=\left(f_{x1}^{b1}+f_{x2}^{b2}\right)/2$$

(21) $$f_{y}^{a}=\left(f_{y1}^{b1}+f_{y3}^{b3}\right)/2$$

(22) $$f_{z}^{a}=\left(f_{z2}^{b2}+f_{z3}^{b3}\right)/2$$

#### 3.1.2. Measurement Equation

By analyzing the structure of URMINS, it is obvious that the navigation information output by CSINS1 and CSINS2 comes from the same carrier. Therefore, there are:

(23) $$\begin{array}{c} {\tilde{A}}_{1}=A+\delta A_{1},\;{\tilde{A}}_{2}=A+\delta A_{2}\\ {\tilde{V}}_{1}^{n}=V^{n}+\delta V_{1}^{n},\;{\tilde{V}}_{2}^{n}=V^{n}+\delta V_{2}^{n}\\ {\tilde{p}}_{1}=p+\delta p_{1},\;{\tilde{p}}_{2}=p+\delta p_{2} \end{array}$$

The symbols $A,\;\;V^{n}$ and ***p***, respectively, represent the actual values of the carrier’s attitude, velocity, and position, and the specific expressions are shown in Equation (24).

(24) $$A={\left[\theta \;\gamma \;\psi \right]}^{T},V^{n}={\left[V_{E}^{n}\;V_{N}^{n}\right]}^{T},p={\left[L\;\lambda \right]}^{T}\;$$

The symbols ${\tilde{A}}_{1}$, $\delta A_{1}$, ${\tilde{A}}_{2}$ and $\delta A_{2}$ represent the attitude and attitude error of CSINS1 and CSINS2, respectively. The symbols ${\tilde{V}}_{1}^{n}$, $\delta V_{1}^{n}$, ${\tilde{V}}_{2}^{n}$, $\delta V_{2}^{n}$, ${\tilde{p}}_{1}$, $\delta p_{1}$, ${\tilde{p}}_{2}$, $\delta p_{2}$ have the similar definition as the symbols of attitude.

The difference between the output information of CSINS1 and CSINS2 can be obtained by Equation (25). The acceleration and angular velocity caused by the carrier’s motion are not included in the measured values, so this method can estimate the installation error in a dynamic environment.

(25) $$\delta {\tilde{A}}_{12}={\tilde{A}}_{1}-{\tilde{A}}_{2},\;\delta {\tilde{V}}_{12}^{n}={\tilde{V}}_{1}^{n}-{\tilde{V}}_{2}^{n},\delta {\tilde{p}}_{12}={\tilde{p}}_{1}-{\tilde{p}}_{2}\;$$

The measurement equation of the system is as follows.

***Z****(t)*=***H****(t)****X****(t)*+***V****(t)* (26)

where ***Z****(t)* represents the measurement vector of the system. ***H****(t)* is the measurement matrix of the URMINS. ***V****(t)* is the measurement noise of the system. The detailed expressions of the symbol in Equation (26) are shown in Equations (27)–(30).

(27) $$Z\left(t\right)=\left[\delta {\tilde{A}}_{12}\;;\;\delta {\tilde{V}}_{12}^{n}\;;\;\delta {\tilde{p}}_{12}\right].$$

(28) $$H\left(t\right)=\left[\begin{array}{cc} C_{\phi }^{A} & 0_{3\times 2}\\ 0_{2\times 3} & I_{2}\\ 0_{2\times 3} & 0_{2\times 2} \end{array}\;\;\begin{array}{cc} 0_{3\times 2} & 0_{3\times 6}\\ 0_{2\times 2} & 0_{2\times 6}\\ I_{2} & 0_{2\times 6} \end{array}\right]\;$$

(29) $$C_{\phi }^{A}=\left[\begin{array}{ccc} -cos\psi & -sin\psi & 0\\ sin\psi /cos\theta & -cos\psi /cos\theta & 0\\ -sin\psi tan\theta & cos\psi tna\theta & -1 \end{array}\right]$$

(30) $$I_{2}=[\begin{array}{cc} 1 & 0\\ 0 & 1 \end{array}]$$

In this paper, to further demonstrate the effectiveness of the Kalman filter algorithm, observability analysis of the state variables in Kalman filter is essential. Here the attitude, velocity, and position errors $\phi_{12}$, $\delta V_{12}$, and $\delta p_{12}$ are obtained from $\delta {\tilde{A}}_{12}$, $\delta {\tilde{V}}_{12}^{n}$, and $\delta {\tilde{p}}_{12}$, respectively, so these navigation error terms are entirely observable. Analysis of Equations (18) and (19) shows that the installation error term $\Delta \mu_{k}^{i}$ is related to the measurement value of the accelerometer, the encoder and the third-axis sensor of each module. Therefore, the linear motion, angular motion and design of the rotation scheme of the carrier can stimulate the installation error and improve the observability of the installation error of the system.

### 3.2. Compensation Scheme

**Figure 4:**
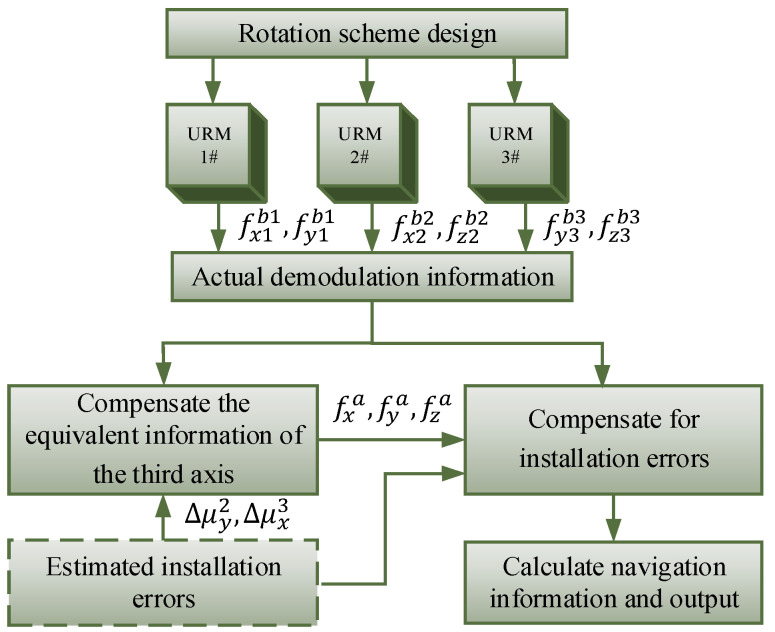
A block diagram of compensating for installation errors.

For the installation error caused by replacing modules of URMINS, the block diagram of the installation error compensation method proposed in this paper is shown in Figure 4.

The rotation scheme of a URM needs to be designed, and the motor rotary speed of each module is set to 6°/s to achieve continuous rotation in one direction. Each module’s inertial sensor measurement data are demodulated, and the sensor data, called actual demodulation information, is obtained in the navigation solution module. Firstly, installation errors $\Delta \mu_{y}^{2}$ and $\Delta \mu_{x}^{3}$ are compensated to get the equivalent information $f_{x}^{a}$, $f_{y}^{a}$ and $f_{z}^{a}$ along the rotation axis of each module. Among them, the calculation method of the equivalent information is as Equations (20)–(22). Equations (31) and (32) show the compensated error terms. Secondly, the installation error between modules associated with URM 2# and URM 3# is compensated by Equations (18) and (19), and the sensor’s data under the *b*-frame after compensation is obtained. Finally, since the accuracy of the sensors of each module is the same, the average value is taken as the data output of each axis on the *b*-frame, and the traditional algorithm of SINS is used to calculate the navigation information.

(31) $$\begin{array}{c} \delta f_{x2}^{c}=-\Delta \mu_{y}^{2}sin\theta_{2}f_{x2}^{a}+\Delta \mu_{y}^{2}cos\theta_{2}f_{z2}^{a}\\ \delta f_{z2}^{c}=-\Delta \mu_{y}^{2}cos\theta_{2}f_{x2}^{a}-\Delta \mu_{y}^{2}sin\theta_{2}f_{z2}^{a} \end{array}$$

(32) $$\begin{array}{c} \delta f_{y3}^{c}=-\Delta \mu_{x}^{3}sin\theta_{3}f_{y3}^{a}-\Delta \mu_{x}^{3}cos\theta_{3}f_{z3}^{a}\\ \delta f_{z3}^{c}=\Delta \mu_{x}^{3}cos\theta_{3}f_{y3}^{a}-\Delta \mu_{x}^{3}sin\theta_{3}f_{z3}^{a}\; \end{array}$$

We explain the error compensation method in detail. The error compensation process is further introduced by taking the error $\delta f_{x2}^{b}$ of the accelerometer of URM 2# as an example. The error terms presented by substituting URM 2# are shown in Equation (18). The algorithm in Section 2 can estimate the installation error $∆\mu_{k}^{2}$, and the encoder measures the rotation angle $\theta_{2}$. $f_{x2}^{a}$ and $f_{z2}^{a}$ can be measured by two accelerometers mounted in the URM 2#. The unknown quantity $f_{y}^{a}$ needs to be supplied from URM 1# or URM 3# as shown in Equation (34). Here, the installation error caused by $\Delta \mu_{x}^{3}$ needs to be compensated first, as shown in Equation (33). Furthermore, the error caused by the replacement of URM 2# can be paid by Equation (34). The error compensation method of the remaining axis is similar to the above procedure and will not be described in detail.

(33) $$f_{y3}^{b}=f_{y3}^{b3}-\delta f_{y3}^{c}$$

(34) $$f_{y}^{a}=\left(f_{y1}^{b1}+f_{y3}^{b}\right)/2$$

## 4. Simulation Results and Analysis

### 4.1. Simulation of Carrier Motion

Simulation experiments are implemented to verify the effectiveness of the installation error calibration method in Section 3.

**Figure 5:**
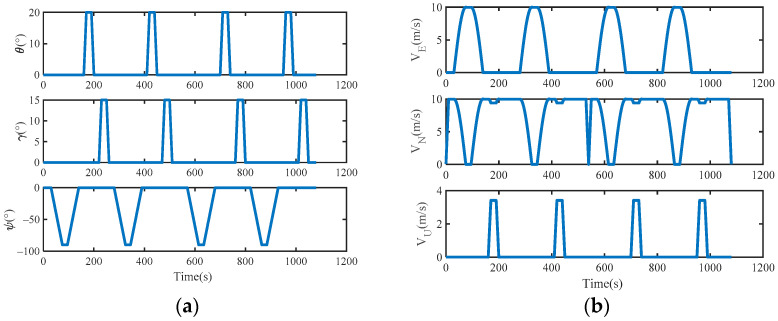
(**a**) The attitude of the carrier; (**b**) the velocity of the carrier.

**Figure 6:**
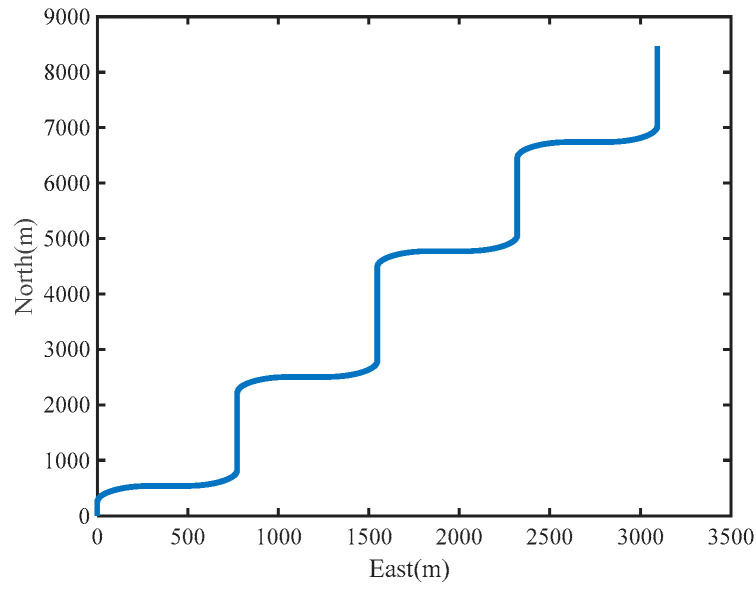
The trajectory of the carrier.

The URMINS is mounted on moving carriers to effectively excite the calibration error to calibrate installation errors between modules. We consider the carrier’s linear and angular motion, the simulated carrier’s attitude, velocity, and trajectory change with time, as shown in Figure 5 and Figure 6. In Figure 6, the starting point of the carrier is at the origin of the coordinates.

### 4.2. Calibration Results of Installation Errors

The carrier motion in Section 4.1 is used as an incentive for self-calibration of the mounting error. The parameters used in the simulation, including inertial sensors’ errors and mounting errors, are shown in Table 1. The method to simulate inertial sensors’ data without noise was described in reference [35]. Here, inertial sensors’ errors are simulated with noise models such as Gaussian white noise, random constant deviation and first-order Markov process. The sensor data used in the simulation are obtained by summing the noise-free raw data with the noisy data. This paper assumes that the *b*1-frame of URM 1# overlaps with the *b*-frame, then the installation error terms $∆\mu_{x}^{1},$ $∆\mu_{y}^{1}$, and $∆\mu_{z}^{1}$ are set to zero. The initial alignment errors of CSINS1 and CSINS2 are the same, as shown in Table 2.

**Figure 7:**
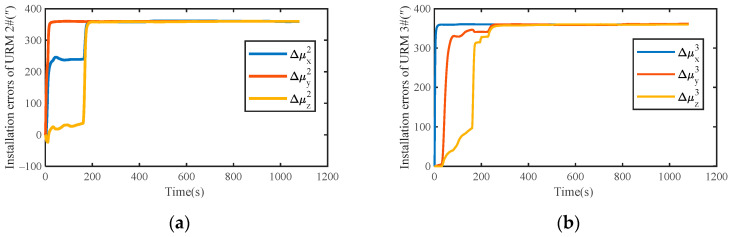
(**a**) Simulation results of installation errors for URM 2#; (**b**) simulation results of installation errors for URM 3#.

**Figure 8:**
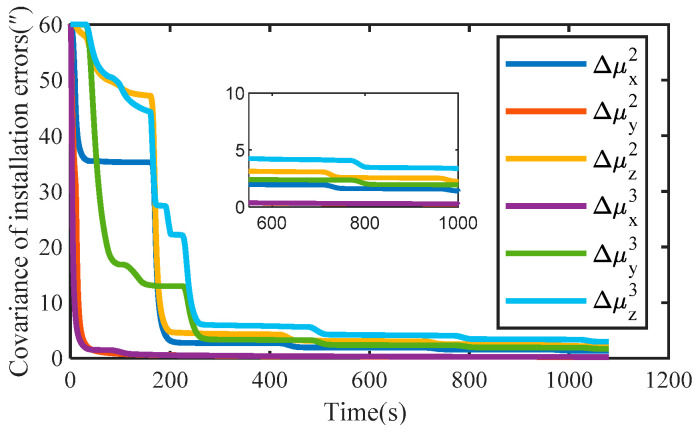
The covariance of installation errors is obtained by simulation.

Assuming that the motor of each module is locked in a fixed position, the installation errors of URM 2# and URM 3# obtained by simulation are shown in Figure 7a,b, respectively. With the increase in simulation time, installation errors obtained by simulation present a stable trend. The covariance curves of installation error are shown in Figure 8. As shown in Figure 8, after the simulation time is 600 s, the covariance values of all installation errors are within 5″. Therefore, the installation error can be estimated quickly and accurately using the self-calibration method proposed in this paper.

Furthermore, the effectiveness of the proposed calibration method was verified, and the installation error was simulated five times. The mean value of installation errors was calculated after 600 s, and the mean value and residual of installation errors were presented in Table 3. The maximum value of the residuals of all installation errors is 1.54″, which further verifies the algorithm’s reliability.

### 4.3. Compensation Results and Analysis

**Figure 9:**
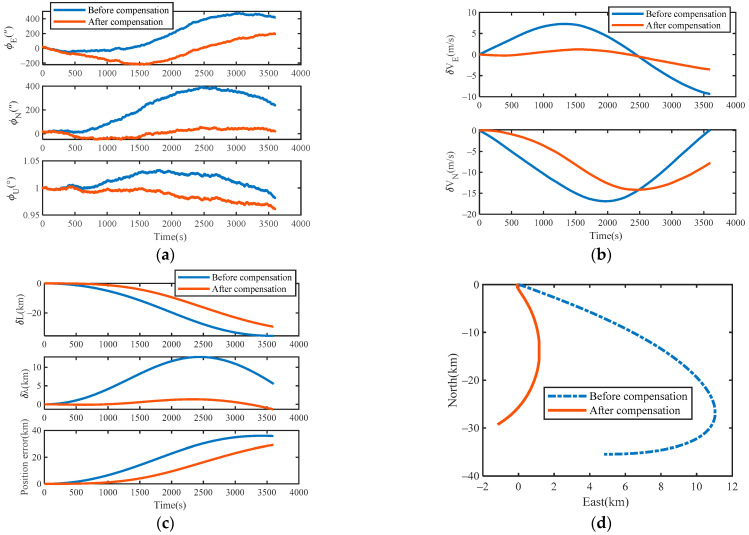
(**a**) Simulation results of attitude errors; (**b**) simulation results of velocity errors; (**c**) simulation results of position errors; (**d**) compare the trajectory of the carrier.

The evaluation method, called true root mean square (TRMS), was used to evaluate the accuracy of the URMINS. The attitude, velocity, and position error curves before and after compensating for the installation error are shown in Figure 9a–c. The simulation data were produced based on MATLAB for one hour under the static condition of the carrier. The motor speed of each module was set to 6°/s. It can be seen from the simulation results of attitude angle errors in Figure 9a that, compared with before compensating for the installation error, the error of the misalignment angles is suppressed after compensating for the installation error. It can be seen from Figure 9b,c that after compensating for the installation error, both the speed errors and the position errors are significantly reduced. The positioning results before and after compensating for URMINS installation errors are shown in Figure 9d to demonstrate the advantages of positioning results after compensating for installation errors. After the installation error of URMINS is compensated, the positioning accuracy is higher.

To verify the validity of the installation error compensation method of the URMINS, the TRMS values of the five simulation results, including the attitude angle error, velocity error and position error, are listed in Table 4. It can be seen from Table 4 that the heading angle accuracy of the URMINS is improved by 2.61%, and the position accuracy of the URMINS is improved by 34.18%. In a word, the installation errors compensation method proposed in this paper can compensate for the installation errors of the URMINS without relying on external information in a dynamic environment. At the same time, the navigation accuracy of the URMINS can be improved.

## 5. Experimental Results and Analysis

### 5.1. Experimental Results of Self-Calibration

**Figure 10:**
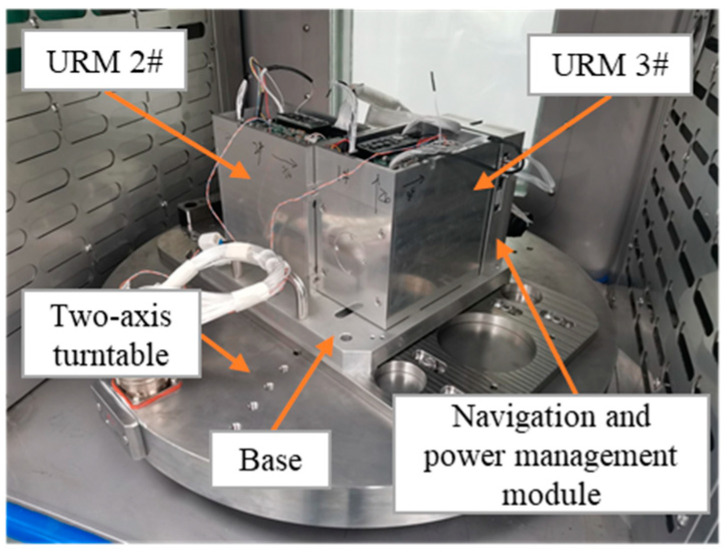
A prototype is used in the experiment.

An experiment with the proposed method was performed using the URMINS fabricated by our laboratory to demonstrate the effectiveness of the self-calibration method in practical applications. In this paper, two modules are used to form URMINS to achieve self-calibration, and the experimental prototype is shown in Figure 10. A computer collected the data output by the URMINS at 100 Hz. The self-calibration method proposed in this paper does not depend on external references, such as turntable and GPS. It only needs the carrier to be dynamic to stimulate the installation error. To verify the effectiveness of the proposed self-calibration algorithm, we installed the URMINS on a dual-axis turntable. The turntable was controlled to provide a sway randomly motion for the URMINS, simulating the angular motion of the carrier. The installation error is estimated by applying a self-calibration algorithm. The two-axis turntable used in the experiment is 2TD-550 fabricated by the Jiujiang Precision Test Technology Research Institute, and the specific parameters of this turntable are shown in Table 5.

**Figure 11:**
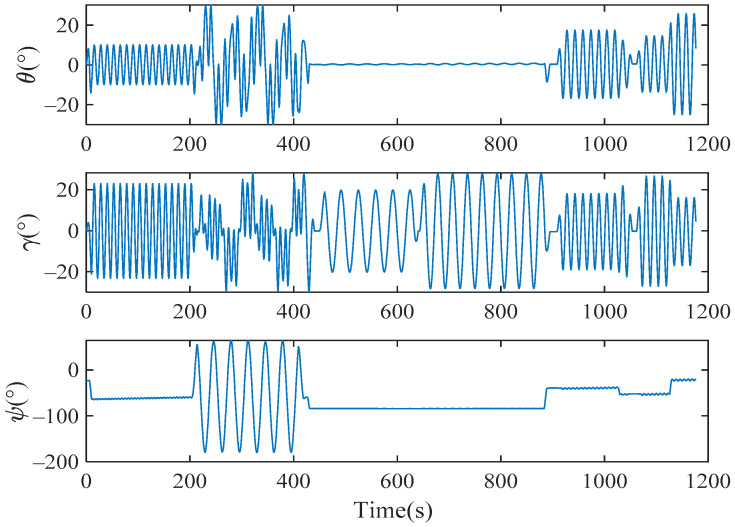
The attitude of the carrier was simulated by the sway of the turntable.

Here, the CSINS1 consists of $X_{b2}$, $Z_{b2}$ of URM 2#, and $Y_{b3}$ of URM 3#, while $X_{b2}$, $Z_{b3}$ of URM 3#, and $X_{b2}$ of URM 2# constitute the CSINS2. The structure of each module is consistent with that described in Section 2.1. The difference in navigation information of CSINS1 and CSINS2 was used as the measurement value to estimate the installation errors between modules. Assuming that the *b*-frame overlapped with the *b*2-frame of URM 2#, the installation error $\Delta \mu_{k}^{2}$ is zero. Reinstall URM 3# and use the dynamic self-calibration method proposed in this paper to estimate the installation error $\Delta \mu_{k}^{3}$. The motor of each module was controlled to stop at a position where the angle information output by the encoder is zero. The turntable is controlled to sway randomly to simulate the random motion of the carrier. The attitude of the carrier is shown in Figure 11.

The data along the $Z_{b}$-axis of the carrier were obtained through URM 2# and URM 3# by this experimental prototype, and only the information along the $Z_{b}$-axis was redundant. These data along the $X_{b}$-axis and $Y_{b}$-axis were reused in CSINS1 and CSINS2. Therefore, by further analyzing the error term $\delta f_{z3}^{b}$ of Equation (19), it can be concluded that installation errors $\Delta \mu_{x}^{3}$ and $\Delta \mu_{y}^{3}$ are fully observable, while $\Delta \mu_{z}^{3}$ is unobservable.

**Figure 12:**
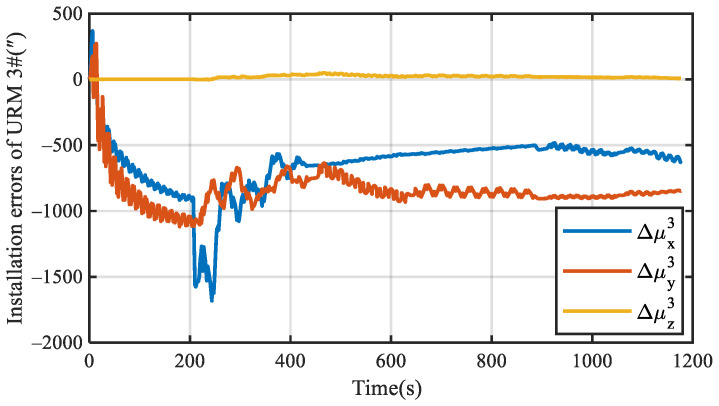
Experimental results of installation errors for URM 3#.

**Figure 13:**
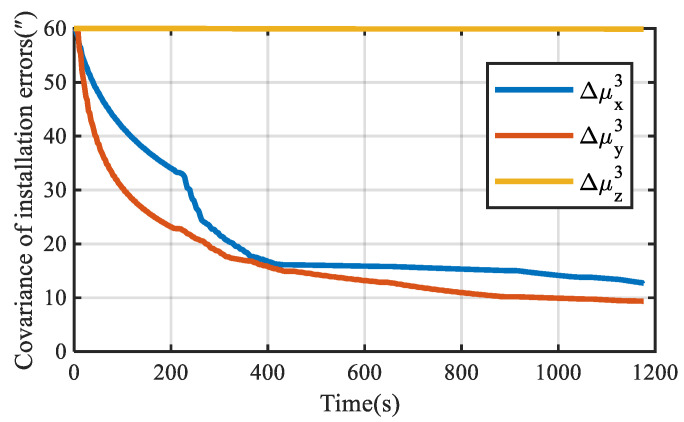
Experimental results of installation errors’ covariance.

The experimental results of the installation error for URM 3# are shown in Figure 12. $\Delta \mu_{x}^{3}$ and $\Delta \mu_{y}^{3}$ tend to be stable over time, while $\Delta \mu_{z}^{3}$ cannot be estimated. The covariance curve of the installation error is shown in Figure 13, which also proves that $\Delta \mu_{z}^{3}$ is unobservable, and $\Delta \mu_{x}^{3}$ and $\Delta \mu_{y}^{3}$ are gradually converging over time. The mean value of the installation error was calculated after 800 s, and the mean values of $\Delta \mu_{x}^{3}$ and $\Delta \mu_{y}^{3}$ were −544.72” and −871.52”, respectively. Therefore, the experimental results agree with the theoretical analysis, which proves the effectiveness of the proposed self-calibration method.

### 5.2. Compensation Results and Analysis

**Figure 14:**
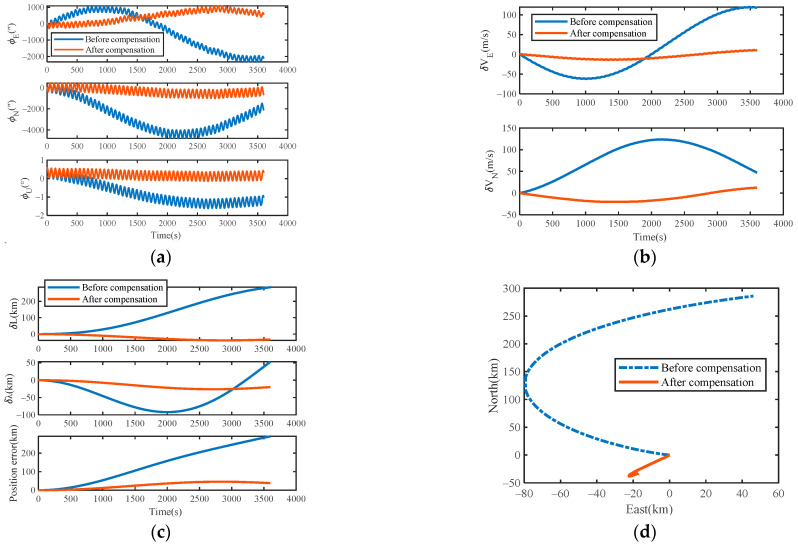
(**a**) Experimental results of attitude errors; (**b**) experimental results of velocity errors; (**c**) experimental results of position errors; (**d**) compare the trajectory of the carrier.

Compensate for the installation error of URMINS with the estimated mean values of $\Delta \mu_{x}^{3}$ and $\Delta \mu_{y}^{3}$. Since $\Delta \mu_{z}^{3}$ cannot be calculated, this value is set to zero. The experimental data were collected for one hour under the static condition of the carrier. The motor’s speed of each module was set to 5.32°/s. From the experimental results of the navigation errors in Figure 14a–c, it is found that the attitude angle error, velocity error and position error are suppressed after compensating for the installation error compared to before compensating for the installation error. The positioning results before and after compensating for the URMINS installation error are shown in Figure 14d to further demonstrate the advantages of the positioning results after compensating for the installation error. The positioning accuracy is higher after compensating for the installation error of URMINS.

To digitally present the improvement effect of navigation accuracy, the experimental results, including the TRMS values of attitude angle error, velocity error, and position error, are statistics in Table 6. Among the navigation error parameters, the accuracy improvement range of the eastward misalignment angle is the lowest at 53.51%, and the suppression ranges of the other navigation error parameters are higher than this value. It can be seen from Table 6 the heading angle accuracy of the URMINS was enhanced by 73.12%, and the position accuracy of the URMINS was improved by 81.19%. In conclusion, the experimental results demonstrate that the installation error compensation method proposed in this paper can compensate for the installation error of URMINS without relying on external information and improve the navigation accuracy.

## 6. Conclusions

This paper presents a new self-calibration and compensation method for URMINS installation error, which can estimate the installation error caused by the replacement of a faulty module without additional equipment and significantly improve the navigation accuracy of the system. In this paper, the installation error model of URMINS was established and analyzed. Then, three URMs were combined with URMINS, and the system was designed to output two sets of navigation information. The difference in attitude, speed, and position between two groups of CSINS was taken as the measurement value of the Kalman filter, and the installation error between modules was estimated. After demodulation, the average value of redundant information was taken to calculate the carrier’s attitude, velocity, and position. The simulation and experimental results show that the installation errors caused by module replacement can tend to the exact value with this method. Compared with the static navigation results without compensating for the installation error, both simulation and experiment prove that the carrier’s attitude, speed, and position accuracy significantly improve after paying for the installation error. The experimental results show that after compensating for the installation error, the heading angle accuracy and horizontal positioning accuracy of URMINS are improved by 73.12% and 81.19%, respectively. The self-calibration method proposed in this paper can be applied in a real situation where the carrier is in a dynamic environment and the inertial sensor has information redundancy. The estimation of the installation error can be realized without relying on the external reference. In addition, the URMINS is easy to maintain and can suppress errors in pure INSs, which provides convenience for carriers to achieve long-term, high-precision navigation in concealed environments.

## Figures and Tables

**Table 1:** Set the errors used in the simulation.

Inertial Sensors Errors	Value	Inertial Sensors Errors	Value
Gyroscope constant drift	0.02 °/h	Accelerometer constant bias	50 μg
Gyroscope scale factor	50 ppm	Accelerometer scale factor	50 ppm
Gyroscope angle random walk	0.02°/√h	Accelerometer random walk	5 μg/√Hz
URM 2# orthogonal installation error $∆\mu_{k}^{2}$	$360^{\prime \prime }$	URM 3# orthogonal installation error $∆\mu_{k}^{3}$	$360^{\prime \prime }$

**Table 2:** Initial alignment errors.

Errors	Value	Errors	Value
Pitch error (″)	20	Velocity error (m/s)	0.01
Roll error (″)	20	Position error (m)	0.1
Yaw error (°)	1		

**Table 3:** The installation errors and residuals were obtained by simulation.

	$\mu_{x}^{2}(\prime \prime )$	$\mu_{y}^{2}(\prime \prime )$	$\mu_{z}^{2}(\prime \prime )$	$\mu_{x}^{3}(\prime \prime )$	$\mu_{y}^{3}(\prime \prime )$	$\mu_{z}^{3}(\prime \prime )$
1	361.86	359.19	359.08	359.73	357.42	357.62
2	360.03	359.90	358.70	360.67	359.71	359.40
3	358.21	360.05	361.03	359.93	362.16	358.63
4	367.11	360.27	359.82	361.17	353.90	359.95
5	359.88	359.51	359.23	360.20	359.13	359.45
Average	361.42	359.79	359.57	360.34	358.46	359.01
Residual	−1.42	0.21	0.43	−0.34	1.54	0.99

**Table 4:** Simulation results of attitude angle error, velocity error, and position error.

Navigational Errors	Before Compensation	After Compensation	Improvement Range (%)
$\phi_{E}\;\left({}^{\circ}\right)$	0.07	0.04	48.60
$\phi_{N}\;\left({}^{\circ}\right)$	0.06	0.01	85.29
$\phi_{U}\;\left({}^{\circ}\right)$	1.00	0.97	2.61
$\delta V_{E}\;\left(m/s\right)$	4.08	1.62	60.35
$\delta V_{N}\;\left(m/s\right)$	11.15	9.87	11.51
δL (km)	21.04	13.97	33.60
δλ (km)	5.54	2.89	47.78
δP (km)	21.59	14.21	34.18

**Table 5:** Parameters of the two-axis turntable.

Parameters	Value	Parameters	Value
Angular position error	≤2″	Angular rate error	≤0.002°/s
Rotational error of shafting	≤2″	Angular rate range of the inner frame	±0.001~±500°/s
Shafting perpendicularity error	≤2″	Angular rate range of the outer frame	±0.001~±300°/s

**Table 6:** Experimental results of attitude angle error, velocity error, and position error.

Navigational Errors	Before Compensation	After Compensation	Improvement Range (%)
$\phi_{E}\;\left({}^{\circ}\right)$	0.34	0.16	53.51
$\phi_{N}\;\left({}^{\circ}\right)$	0.86	0.14	84.28
$\phi_{U}\;\left({}^{\circ}\right)$	1.00	0.27	73.12
$\delta V_{E}\;\left(m/s\right)$	65.78	8.81	86.60
$\delta V_{N}\;\left(m/s\right)$	87.60	13.48	84.61
δL (km)	154.54	26.15	83.08
δλ (km)	57.73	18.12	68.62
δP (km)	162.25	30.52	81.19

## Data Availability

The data that support the findings of this study are available from the corresponding author upon reasonable request.

## Appendix A

The accelerometers’ output errors of the *X*-axis and *Z*-axis in the *b*-frame due to the absence of the *Y*-axis are shown in Equation (6). Here, we only consider the primary term of the installation error. In the *b*2-frame, the measured value of the accelerometer is $f^{b2}$.

(A1) $$f^{b2}=C_{s20}^{b2}C_{s2}^{s20}C_{a2}^{s2}f^{a2}$$

And then, the transformation matrices in Equation (A1) are written as follows.

(A2) $$C_{a2}^{s2}=\left[\begin{array}{ccc} 1 & -\beta_{xz}^{2} & \beta_{xy}^{2}\\ \beta_{yz}^{2} & 1 & -\beta_{yx}^{2}\\ -\beta_{zy}^{2} & \beta_{zx}^{2} & 1 \end{array}\right]$$

(A3) $$C_{s2}^{s20}=\left[\begin{array}{ccc} cos\theta_{2} & 0 & sin\theta_{2}\\ 0 & 1 & 0\\ -sin\theta_{2} & 0 & cos\theta_{2} \end{array}\right]$$

(A4) $$C_{s20}^{b2}=\left[\begin{array}{ccc} 1 & -\mu_{z}^{2} & \mu_{y}^{2}\\ \mu_{z}^{2} & 1 & -\mu_{x}^{2}\\ -\mu_{y}^{2} & \mu_{x}^{2} & 1 \end{array}\right]$$

In transformation matrix $C_{a2}^{s2}$, the installation error angles $\beta_{yz}^{2}$ and $\beta_{yx}^{2}$ are zero since no sensor is mounted along the $Y_{a2}^{2}$-axis. The rotation angle of the motor is indicated by the symbol $\theta_{2}$. The vector $f^{a2}$ represents the measurement of the accelerometer installed in URM 2# in the *a*2-frame, where there exists $f^{a2}={\left[f_{x2}^{a}\;0\;f_{z2}^{a}\right]}^{T}$.

The accelerometers’ output errors of the *Y*-axis and *Z*-axis in the *b*-frame due to the absence of the *X*-axis are shown in Equation (7). Here, we only consider the primary term of the installation error. In the *b*3-frame, the measured value of the accelerometer is $f^{b3}$.

(A5) $$f^{b3}=C_{s30}^{b3}C_{s3}^{s30}C_{a3}^{s3}f^{a3}$$

And then, the transformation matrices in Equation (A5) are written as follows.

(A6) $$C_{a3}^{s3}=\left[\begin{array}{ccc} 1 & -\beta_{xz}^{3} & \beta_{xy}^{3}\\ \beta_{yz}^{3} & 1 & -\beta_{yx}^{3}\\ -\beta_{zy}^{3} & \beta_{zx}^{3} & 1 \end{array}\right]$$

(A7) $$C_{s3}^{s30}=\left[\begin{array}{ccc} 1 & 0 & 0\\ 0 & cos\theta_{3} & -sin\theta_{3}\\ 0 & sin\theta_{3} & cos\theta_{3} \end{array}\right]$$

(A8) $$C_{s30}^{b3}=\left[\begin{array}{ccc} 1 & -\mu_{z}^{3} & \mu_{y}^{3}\\ \mu_{z}^{3} & 1 & -\mu_{x}^{3}\\ -\mu_{y}^{3} & \mu_{x}^{3} & 1 \end{array}\right]$$

In transformation matrix $C_{a3}^{s3}$, the installation error angles $\beta_{xz}^{3}$ and $\beta_{xy}^{3}$ are zero since no sensor is mounted along the $X_{a3}^{3}$-axis. The rotation angle of the motor is indicated by the symbol $\theta_{3}$. The vector $f^{a3}$ represents the measurement of the accelerometer installed in URM 3# in the *a*3-frame, where there exists $f^{a3}={\left[0\;f_{y3}^{a}\;f_{z3}^{a}\right]}^{T}$.
